# Abnormality of visual neuromagnetic activation in female migraineurs without aura between attacks

**DOI:** 10.1186/s10194-018-0957-9

**Published:** 2019-01-16

**Authors:** Zhi Y. Zhou, Yan W. Yu, Di Wu, Hong X. Liu, Jing Xiang, Ting Wu, Qi Q. Chen, Xiao S. Wang

**Affiliations:** 1 The Department of Neurology, Nanjing Brain Hospital, Nanjing Medical University, Guang Zhou Road 264, Nanjing, Jiangsu 210029 People’s Republic of China; 2 The MEG Center, Division of Neurology, Cincinnati Children’s Hospital Medical Center, 3333 Burnet Avenue, Cincinnati, OH 45220 USA; 3 The MEG Center, Nanjing Brain Hospital, Nanjing, Jiangsu People’s Republic of China

**Keywords:** Migraine, Visual evoked magnetic fields, Magnetoencephalography

## Abstract

**Objective:**

The present study aimed to preliminary explore the abnormal neuromagnetic activation in female migraine patients between attacks using magnetoencephalography (MEG) and pattern reversed visual evoked magnetic fields (PR-VEFs).

**Methods:**

A total of 17 female migraine subjects during the headache-free phase and 17 healthy controls (HC) were studied using a 275-channel magnetoencephalography (MEG) system. In this study, visual evoked magnetic fields (VEFs) were generated by a pattern-reversal check as the visual stimulus. The average of 100 VEFs was evolved by different half patterns were averaged and used to analyze waveform, spectrum, and source location within two frequency ranges (5–100 and 100–1000 Hz), respectively.

**Results:**

In migraine subjects, the latency of second peak of VEFs (VIIs) showed significant prolongations when compared with HC. On the sensor level, the cortical spectral power in migraine subjects was similar to that of HC in the 5–100 Hz range and was lower in the 1000–1000 Hz range. There was a decrement of source strength in the visual cortex in migraine patients when compared to HC in both the 5–100 and 100–1000 Hz frequency range. Moreover, there was a similar odds of activation in 5–100 and 100–1000 Hz frequency ranges in the area beyond the primary visual cortex between the two groups. In addition, no correlation was observed between clinical data (intensity of headache, headache-history duration, the frequency of headaches) and MEG results.

**Conclusions:**

The findings presented in the current study, suggested that interictal cortical activation following a visual stimulus was low in female migraine patients. The low pre-activation was detected in the visual cortex using VEF and MEG in both low and high-frequency band. Our results add to the existing evidence that cortical interictal excitability change may be relative to the pain-module mechanism in migraine brains. Thus, our data improved the apprehension of the cortical disorder of migraine in the high-frequency domain.

## Background

Migraine is a common, disabling neurological disorder, clinically manifested by episodes of moderate to severe episodic pain that are accompanied by autonomic nervous system dysfunction [1]. As one of the 20 most disabling diseases [2], migraine exerts a huge impact on individuals and society, and is considered an important public health problem. It has been estimated that approximately 31% of migraine patients have missed at least 24 h of work or school in the latest 3 months because of migraine, and 51% of patients has reported at least a 50% reduction in productivity at work or school [3]. The prevalence of migraine is different between the male and female population, and according to multiple surveys, the prevalence of migraine in females is 3-fold higher compared to that in males in the general population [4, 5].

Migraine has been considered a primary disorder of the brain, not a primary vascular event [1, 6]. Recent reports have shown that dysfunctions of the cerebral cortex and brain-stem or diencephalic are fundamental in the underlying mechanism of migraine [6, 7]. Among many cortical regions, the occipital cortex has been an obvious region of interest regarding the functional change in the brain of a migraine patient for many reasons. Firstly, approximately 30% of migraineurs have transient neurological symptoms that are most frequently visual (MA) preceding headache attacks [8], second, most (60%) migraineurs suffer from distinctive visual disturbance before or during a headache attack [9]. In addition, exposing interictal migraineurs to light may trigger the initialization of a headache [10]. Albeit the essential role the visual cortex may plat in the pathophysiology of migraine, electrophysiological experiments have failed to consistently conclude on the changes in excitability of migraine brains throughout the periodic stage of migraine [11, 12], the cerebral mechanisms underlying such differences remain unclear.

The visual evoked potential (VEP) is an acknowledged neurologic diagnostic tool for detecting disease-induced changes in visual pathways or visual cortical function. VEP studies have a domesticated deficiency in cortical habituation ability in individuals with migraines, which is evidenced by failing to decrement in amplitude over time [13, 14], and shows cortical hyperresponsivity of the visual cortex. Magnetoencephalography (MEG, an atraumatic neuroimaging modality for investigating functional activation in the brain with outstanding temporal resolution and satisfactory special resolution [15]. MEG receives neuromagnetic signals that penetrate the skull and scalp without distortion caused by the skull, skin, or other tissues [16]. Compatible with the triphasic deflection in VEPs, neuromagnetic activation elicited by the visual stimulus can be detected by MEG [11, 17]. Among the components of PR-Visual Evoked Fields (VEF), P100 was considered the most reliable, robust activation, and therefore is widely investigated in VEF/VEP studies [18, 19]. According to the anatomical organization of the visual cortex, the left striate cortex or ex-striate cortex is activated by the stimulus on the right side of the visual field, and vice-versa (known as the Cruciform Model) [20, 21].

Previous reports in both VEP and VEF studies involving migraine subjects have shown conflicting results mainly due to methodological differences (i.e., check size, stimulus fields, reversal rate, etc.), and the patient’s state during the experiment (e.g., the time interval to last attack, menstrual phase) [12, 22]. Unfortunately, these studies only focused on the waveform level in the comparatively low-frequency band (< 100 Hz) [23]. Although high-frequency oscillations may reveal more information about the functional changes in cortical function in the human brain [24–26], the spatiotemporal characteristics of visual evoked neuromagnetic activation in migraine patients on a high-frequency level is mostly unclear.

In this study, we aimed to investigate the signature of aberrant visual evoked neuromagnetic activation on the level of different frequency ranges in female subjects with migraine without aura (MwoA) using MEG and a reversal checkboard pattern (PR-VEFs). To analyze the data, Morlet Wavelet analysis was used, instead of conventional band-pass filters and a waveform-based beamformer to demonstrate the frequency distribution of VEF. Unlike other VEP/VEFs studies, which focused on the central cortical excitability habituation of the migraine brain, we analyzed the visual neuromagnetic spectrograms on the waveform, sensor and source level at two main frequencies: 5–100 Hz, and 100–1000 Hz. We hypothesize that neuromagnetic activation evoked by visual stimuli in the female migraineur was aberrant from that of HC, and this change could be identified at both low (5–100 Hz) and high-frequencies (100–1000 Hz). To our knowledge, this is the first study that focuses on the visual evoked neuromagnetic field in female migraineurs in which MEG and a wide frequency range were used.

## Methodology

### Subjects

Twenty female patients (age: 35.2 ± 6.8 years) from the neurology outpatient department of Nanjing brain hospital (NBH) were enrolled in the present study. Inclusion criteria were as follows (i): Patients explicitly diagnosed with migraine without aura (MwoA) in accordance with the International Classification of Headache Disorders, 3nd Edition bete (ICHD-III beta) [27]; (ii): No medical history of other neurological or ophthalmological disorders (patients as well as HC). (iii) No receipt of any pharmacological prophylactic treatment in 3 months prior to the study, such as valproate, triptans or ergot derivatives). HC matched migraine patients regarding age, gender, and education degree. HC were recruited from employees of the Nanjing brain hospital and their relatives or friends. Inclusion criteria for control group included: (i): Healthy individuals without a history of neuropsychiatric disorders, migraine or any other form of headache, brain injury, and any vision deficiency. Exclusion criteria for both patients and HC included: (i) The presence of any metal implant that might cause magnetic noise in the MEG data; (ii) Inability to remain still during the MEG recording. Migraine subjects were asked to be recorded during the interictal phase (at least 2 days before or after a migraine attack). Clinical characteristics of migraine subjects were accessed by a questionnaire prior to MEG recording, the contents of clinical assessment included: age, disease history, headache frequency (times/month), duration of headache attacks in the latest month, headache locus, and headache accompany symptoms, such as phonophobia, photophobia, nausea and vomiting, intensity of headache assessed by the Visual Analogue Scale (VAS).

### Visual stimulus paradigm

A MEG scan was performed on all respondents while they were asked to stare at a fixed yellow dot in the middle of checkboard pattern on a screen that was placed approximately 32 cm in front of the subjects. Patients were asked to try limit blinking because blinking could result in noise in MEG data. Pattern-reversal checkerboard stimuli were generated using customized software, Brain X (Jing Xiang, Ohio, USA, Cincinnati children hospital) [28]. The visual stimulus consisted of three consecutive reversal patterns with a reversal rate of 1 Hz. These patterns are sequentially presented as: full-field, left-field, and right-field, and the presence of each pattern was 600 ms, with a 400 ms gap between patterns. The size of the checkboard was 60 min of arc, while extended 15(W)*22 (H) in the left hemifield of the subject with the mean luminance set at 12 cd/m^2^, and contrast 0.94. On the screen, there was a delay of 400 ms between trigger onset and stimulus presentation and this time lag was subtracted from the target time window. Visual task stimulus consisted of 100 triggers for each field type (left, right and full field) for one set of recording. The stimulus presentation and signal recording were accomplished with Brain X software, the 100 times of responses from different fields were automatically collected and averaged by the software. Each set lasted for about 5 min, each participant was asked to complete 2 sets of tasks.

### MEG recording

For each participant, the MEG signal was recorded in a controlled magnetic-shielded room by integration of a whole-head 275 channel (CTF, Canada). All patients were requested to remove any magnetic materials from their bodies. There were attachments of electromagnetic coils (Fiducial markers) at the left and right preauricular points and at the nasion point to measure the patients head position in comparison to the sensors of MEG. During the entire scanning procedure, participants were requested to lay in a comfortable position with both arms resting on each side For each channel, MEG record was digitized at 6000 Hz. The acquisition window was set at 1000 ms for each trial, and the 400 ms after the trigger (presence of the reversal pattern) was recorded in the MEG system. MEG data were then recorded by the system after a third-order gradient noise cancellation process. The threshold of permitted head movement during MEG scanning was 5 mm. If head movement during one scan was beyond 5 mm, the dataset was disregarded, and a new scan was recorded.

### Magnetic resonance imaging scan

All participants in this study underwent three-dimensional magnetic resonance imaging (MRI) on a 1.5 MRI (Singa, GE, USA). In brief, three fiduciary marks were placed at locations that were identical to the position of the three coils used in the MEG recordings for the co-registration of the MRI data and MEG data sets. Subsequently, all anatomic landmarks were made identifiable in the MRIs.

## Data analysis

### Morphology

MEG data from all participants were manually analyzed using a custom-designed program, MEG Processor. In brief, data were firstly processed by removal of the Direct Current (DC) base on the results on a pre-trigger baseline. Subsequently, a low pass (5 Hz) and a high pass filter (100 Hz) were applied to obtain the morphology VEFs. The latency of the magnetic fields was measured by putting the cursor on each of the peaks of neuromagnetic components. At least three neuromagnetic components were identified, namely VI, VII, and VIII. In the present study, the quantification of waveforms following the visual stimulus focused on VII.

### Time-frequency analysis

MEG waveforms information was transformed to spectrograms using the Morlet continuous wavelet transform. The average of all trials prior to the transformation was taken, therefore spectrograms are presented as averaged time-frequency domains [29–31]. The spectral characteristics of MEG data were analyzed with spectrograms computed with Morlet continuous-wavelet algorithm using the following equation:

$$ w\left(t,s\right)={C}_{\sigma }{\pi}^{-\frac{1}{4}}{e}^{-\frac{1}{2}{t}^2}\left({e}^{i\sigma t}-{k}_{\sigma}\right) $$

Because frequency-temporal resolution changes with the sigma value, this study dynamically changes sigma value (the number of wave circles) according to frequency ranges [30, 31]. In our previous study, we showed that to achieve a higher time sensitivity in 5–100 Hz and higher frequency sensitivity in 100–1000 Hz range, the sigma values were considered 1, 3 for the 5 to 100 Hz and 100 to 1000 Hz range, respectively, with frequency bins set at 600 Hz [30, 31]. Specifically, neuromagnetic signals below 5 Hz were not included in the analysis because a lengthier time window was required for the computation of low-frequency components of the data, however in the present study, we only focused on time domain in 0–200 ms following the visual trigger, which included all VEF-related components. Neuromagnetic signals above 1000 Hz were also excluded from this study because in our pre-analysis these were not captured at the very-high-frequency range (>1000 Hz). The selection of the frequency ranges was based on our previous findings [29, 31, 32].

### Sensor-level analysis

To better illustrate the source patterns of visual-evoked neuromagnetic activation, the spatial characteristics of visual-evoked neuromagnetic activation at the sensor level were estimated through polarity contour maps [29, 33]. To quantify the strength of activation, the Root-Mean-Square (RMS) value from all sensors was measured to represent the absolute spectral power. All measurements were obtained by a toolkit that automatically obtained the mean and peak value for each frequency bin of all MEG sensors. This approach was employed because the entire calculation could be objectively completed by a computer. Moreover, the quantization spectral power at sensor levels was time-locked at the VIIs activation for all subjects and frequency ranges. For all frequency ranges, the temporal resolution was 6 data points per millisecond. The frequency bands for quantifying spectral power at sensor levels is for 5 to 100 Hz. All spectral power in different frequency bands was normalized mathematically, and the spectral power of a selected region was divided by the number of frequency bins, therefore, the data were comparable with the findings presented in other studies using a different frequency resolution.

### Source analysis

Neuromagnetic sources were localized with volumetric source imaging [34]. In the relatively new method, each coordinate was scanned using a voxel with a 4-mm spatial resolution. In this study, we focused on the VIIs waveform from all subjects because the different components of VEFs have different generators in the visual cortex [35]. Thus, a fixed window was applied for activating VEF. A MEG processor was used for analyzing magnetic sources. According to the cruciform model mentioned above, the dominant activation of VIIs was around the primary visual area (V1), several other visual-related regions may also be activated. The computation was separately performed in two frequency ranges from 5 to 100 Hz and 100 to 1000 Hz.

### Statistical analysis

Latency, spectral power, and source strength of VEFs between migraine subjects and HC were analyzed by Student t-tests. All data were tested by normlity tests (K-S test) before Student t-tests. The relevance between parameters of VEFs and clinical characteristics (intensity of headache, headache-history duration, and the frequency of headache) was analyzed with Pearson correlation test. The difference of odds ratios of activation following visual stimulus in brain areas other than the contralateral primary visual cortex (cPVC) between migraineurs and controls were analyzed by the Fisher exact test. For all participants, statistical analyses were performed twice for the right half and left hemisphere separately. The threshold of statistical significance for differences was set at *p* < 0.05. Bonferroni correction was not applied because there was no multiply comparison for the data.

## Results

### Clinical characteristics

The demography characteristics of migraine patients and HC are shown in Table 1. Out of the 17 patients with migraine, all migraine (100%) subjects suffered from migraine without aura (MwoA), and 9 subjects showed photophobia during the ictal period (the other 8 subjects did not show photophobia during the ictal period). All subjects (100%) were right-handed, 8 subjects (47%) had headache bilaterally; 6 migraine subjects (35%) had unilateral headache attacks on the left supraorbital areas and 3 migraineurs (17%) on the right supraorbital areas. No subjects reported any headache attack triggered by visual stimulus during the recordings.

**Table 1:** Clinical Characteristics of Subjects

Parameters	Migraine	Control
Gender (female/male)	0/17	0/17
Age (years) (mean ± SD)	33.6.2 ± 4.5	32.1 + 3.1
Handedness, right/left	0/17	0/17
Years of migraine (mean ± SD)	9.8 ± 6.4	NA
Frequency of headache per month (mean ± SD)	1.6 ± 1.4	NA
Severity of headache (VAS scale)	6. 6 ± 1.4	NA
Prophylactic treatments in recent three months	0/17	NA
Pain type (number of subjects; multiple descriptions were allowed)		
Throbbing	12	NA
Pressure	2	NA
Constant	3	NA
Sharp	1	NA
Squeezing	2	NA
Stabbing	2	NA
Others	0	NA

Participants may have more than 1 type of pain

*Abbreviations*: *N/A* Not available

### Waveform analysis

**Fig. 1:**
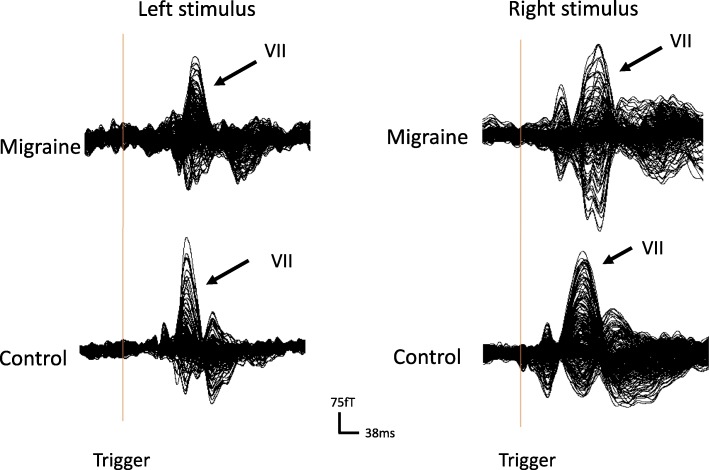
Morphology of waveform of visual-evoked magnetic fields (VEFs) Magnetoencephalography waveforms showing neuromagnetic activation evoked by the visual stimulus (left and right half fields) in a migraine subject (‘Migraine’) and a healthy subject (‘HC’) in a frequency band of 5–100 Hz. There are at least three responses in the visual-evoked magnetic fields (VEFs), respectively “VI”, “VII” and “VIII”. In our study, VII as our observational object. There are differences in morphology of the VII waveforms between migraine subjects and controls. The latencies of the VII in migraine subjects are prolonged when compared with those in the controls. The “Trigger” indicates the start of a visual stimulus

Data were bandpass filtered at 5–100 Hz for demonstration of the waveform. The morphology of waveforms is shown in Fig. 1. In the 5–100 Hz range, the latency of VIIs from migraine subjects was significantly prolonged when compared with HC following both left and right visual stimulus. Significant differences were found between the latency of VIIs evoked by different half stimulus within the migraine group and HC group. Notably, the conventional measurement of amplitudes was not included in this observation because in this study source strength was used to better estimate the activation power.

### Time-frequency analysis

**Fig. 2:**
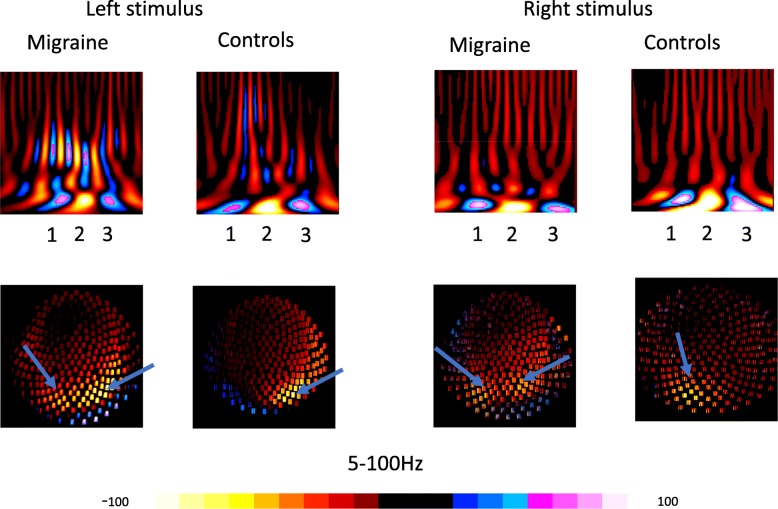
Time-frequency analysis of visual-evoked magnetic fields (VEFs): An example of the results from time-frequency analysis recorded from a migraine subject and a healthy control. Polarity spectrograms and contour maps (second and fourth) of neuromagnetic signals showed a focal activation (lighting color bar) in 5–30 Hz in the low frequency band (50-100 Hz) and 100-300 Hz in the high frequency range during a visual stimulus. There is increased spectral power in the two frequency bands of 100–300 and 500–700 Hz in the polarity spectrograms. The contour maps showed a similar activation pattern of visual evoked magnetic fields (VEF) in migraines and controls

In all migraine subjects (17/17) and all HC (17/17) there were at least three oscillatory components revealed by the polarity spectrograms in the frequency range of 5–100 Hz, especially in 5–30 Hz (see Fig. 2). To quantify the neuromagnetic spectral power, RMS was obtained to access “global spectral power” by calculating the sum of the spectral power from all sensors over the target frequency ranges of 5 to 100 Hz. Interestingly, no significant differences were observed in averaged spectral power in the frequency range of 5–100 Hz between migraine subjects and HC. The source patterns were analyzed with spectral contour maps, which showed that the activation of neuromagnetic responses was localized in the contralateral primary visual cortex in both migraine subjects and HC, and the odds of activation in regions beyond primary visual cortex (i.e. extensive visual cortex; parietal/temporal regions) was similar between migraine subjects and HC in 5-100 Hz. The values of spectrograms from all MEG sensors showed more interindividual variation among migraine subjects when compared to HC.

**Fig. 3:**
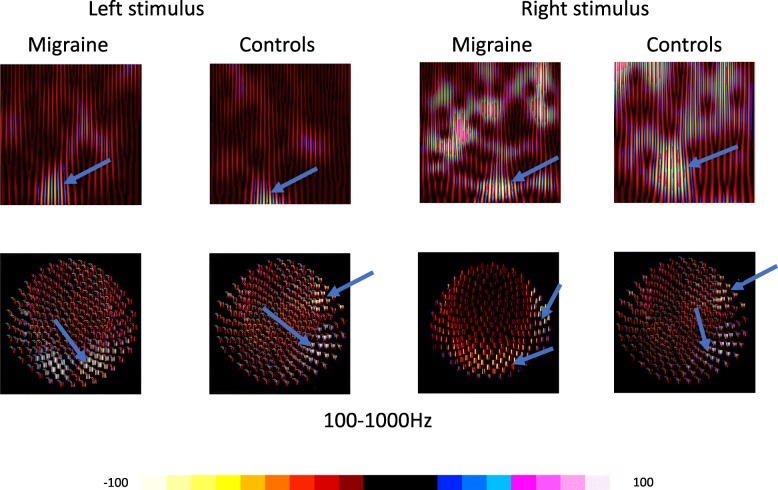
Time-frequency analysis of visual-evoked magnetic fields (VEFs): An example of the results from time-frequency analysis recorded from a migraine subject and a healthy control. Polarity spectrograms and contour maps (second and fourth) of neuromagnetic signals showed a focal activation (lighting color bar) in 5–30 Hz in the low frequency band (50-100 Hz) and 100-300 Hz in the high frequency range during a visual stimulus. There is increased spectral power in the two frequency bands of 100–300 and 500–700 Hz in the polarity spectrograms. The contour maps showed a similar activation pattern of visual evoked magnetic fields (VEF) in migraines and controls

Polarity spectrogram showed focal increased activation in the contralateral visual area represented by the lightning color bar in 100–1000 Hz (see Fig. 3), especially in 100–300 Hz in 9 of the migraine subjects (9/17) and 8 HC (8/17) especially in the 100–300 Hz range. In addition, activation was observed in regions outside of the visual cortex, including the contralateral/ipsilateral temporal lobe. Our data showed that spectral power estimated by RMS in migraine subjects showed a decrement compared to HC in this frequency range, while the odds of activation in these regions was similar between migraine subjects and HC.

### Source analysis

**Fig. 4:**
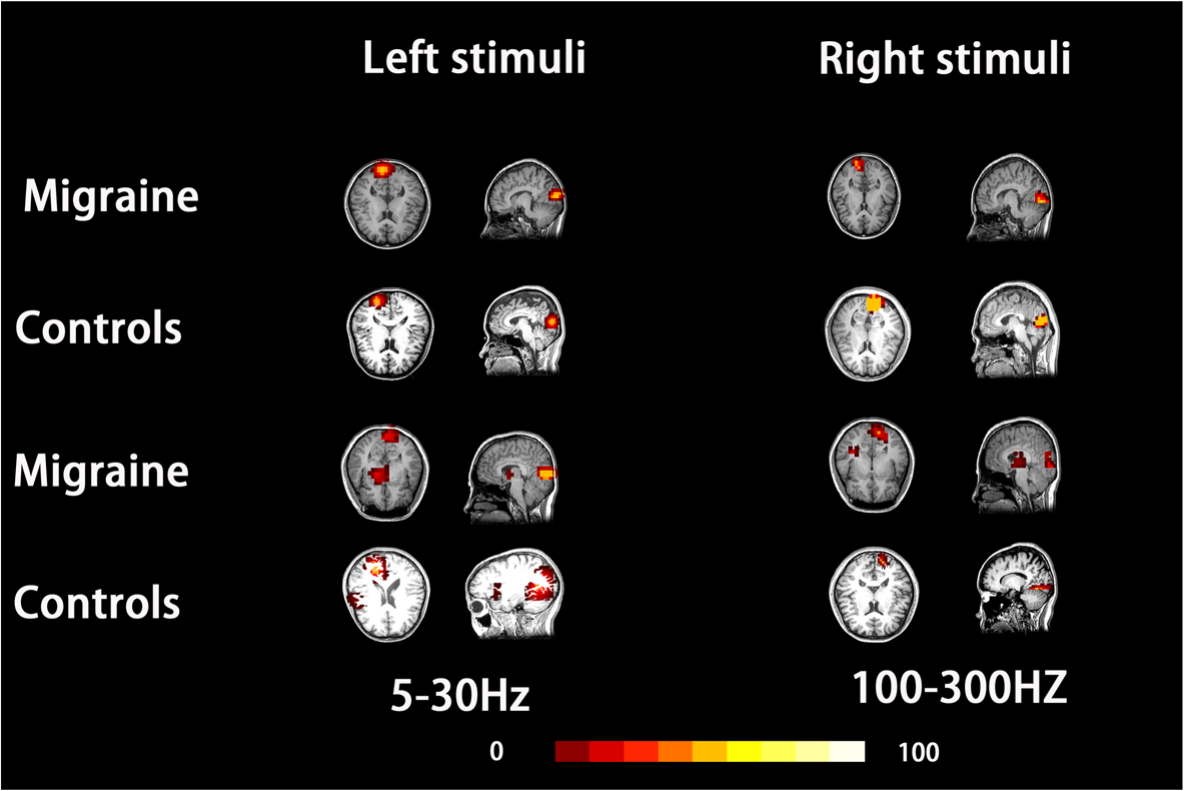
Source location of V2: Magnetic source imaging (MSI) shows the source of activation of neuromagnetic response evoked by a visual stimulus in 5–30 and 100–300 Hz frequency bands in a migraine subject (migraine) and a control (control). The neuromagnetic activation evoked by a visual stimulus is mainly localized in the contralateral primary visual cortex (cPVC) in all migraineurs and controls, activation in regions beyond contralateral primary visual cortex (RBcPVC) was also noticed in some of the migraineurs and controls. Those areas included extensive visual cortex and parietal/temporal areas

In the 5–100 Hz bandpass, all subjects showed activation in the contralateral visual cortex during stimulated by the right-field and left-field stimulus (17/17 vs 17/17). Activation was observed in other brain regions beyond the contralateral primary visual cortex (RBcPVC), these regions distributed from the associated visual cortex to parietal/temporal areas. Among all subjects analyzed using MSI, 7 migraine subjects and 3 HC showed activation in the RBcPVC during the left-field stimulus (8/17 vs 5/17) and 7 migraineurs and 5 C (7/17 vs 5/17) during right-field stimulus (see Fig. 4 and Table 2). No significant differences were observed between the odds of activation in RBcPVC following either right or left visual stimulus (*p* > 0.05). The source strength of activation in migraine subjects was lower when compared to that of HC.

**Table 2:** The main dependent variables analyzed in the MEG spectrograms for migraine subjects and controls

Frequency range	dependent variable	Position of the pattern	Migraine subjects	Healthy controls	*P* value
5-100 Hz	Latency	Left	122.2 ± 4.9	116.2 ± 5.1	0.002
		Right	113.7 ± 5.3	108.8 ± 4.1	0.006
	Spectral Power (RMS)	Left	115.4 ± 11.0	139.5 ± 6.2	<0.00
		Right	92.5. ± 8.9	71.1 ± 8.6	<0.00
	Source Strength (fT/Hz)	Left	355.3 ± 21.8	370.0 ± 16.3	0.035
		Right	258.2 ± 13.5	262.7 ± 10.0	NS
	Location	Left	cPVC: (17/17)	cPVC: (17/17)	NS
			RBcPVC: (8/17)	RBcPVC: (5/17)	NS
		Right	cPVC: (17/17)	cPVC: (17/17)	NS
			RBcPVC: (7/17)	RBcPVC: (5/17)	NS
100-1000 Hz	Source Strength (fT/Hz)	Left	17.4 ± 3.2	21.1 ± 2.7	0.01
		Right	15.1 ± 2.7	17.4 ± 3.2	0.02
	Location	Left	cPVC: (11/17)	cPVC: (9/17)	NS
			RBcPVC:(6/17)	RBcPVC:(7/17)	NS
		Right	cPVC: (11/17)	cPVC: (10/17)	NS
			RBcPVC: (3/17)	RBcPVC: (5/17)	NS

*Abbreviations*: *cPVC* Contralateral primary visual cortex, *RBcPVC* Regions beyond contralateral primary visual cortex, *NS* Not significant

In the 100–300 Hz frequency band, MSI showed that for 11 migraineurs and 9 HC activation was mainly localized in the contralateral primary visual cortex (cPVC) (11/17 vs 9/17) during left-field stimulus, and in 11 migraineurs, and 10 HC during right-field stimulus, while the neuromagnetic activation of cPVC was silent in the remainder of the participants. Moreover, 6 migraineurs and 7 HC (6/17 vs 7/17) showed activation in RBcPVC following left-field stimulus, and 3 migraineurs and 5 HC during the right (3/17 vs 5/17). Similar to that in low-frequency band, the odds of activation in RBcPVC was similar between migraine subjects and HC for both the left and right field. In this frequency band, the strength of activation on the source level in migraine subjects was significantly lower when compared to that of HC following both left and right-field stimulus (*p* < 0.05) (see Fig. 4 and Table 2).

### Neuromagnetic correlations of clinical characteristic

Analysis of the correlation between latency and clinical data revealed that the latency of VIIs activated by left or right-field pattern did not correlated with the frequency of headaches per month, or the severity of headache or the years of migraine. No significant differences were observed between Latency, spectral power (RMS), source strength and these clinical data in either left or right hemisphere.

## Discussion

Our study preliminarily explored the abnormal pattern reversal-visual evoked magnetic fields (PR-VEFs) in female migraine patients during the interictal period in the 5–1000 Hz frequency range. Our data demonstrated that interictal female migraineurs showed aberrant neuromagnetic visual responses that can be detected by MEG and PR-VEFs in both the low and high-frequency range. By adopting measurements of the waveform, spectrogram, and source localization methods in different frequency bands, it was found that the latency of neuromagnetic responses was significantly prolonged when compared to those in HC, while the spectral power regarding cortical activation and odds of activation in other regions besides the primary visual cortex was similar in the low-frequency range (5–100 Hz). In the high-frequency range (100–1000 Hz), however, the activation strength of the neuromagnetic response was lower when compared to that of HC, and the odds of neuromagnetic activation in regions other than contralateral primary visual cortex was similar as in low-frequency range. Our results in the visual neuromagnetic field showed a comparatively normal low-frequency activation and, to a certain degree, aberrant high-frequency oscillation (HFO) in interictal female migraineurs.

The results of MEG waveforms revealed an obvious prolongation in the latency of VII in female migraine patients when compared to HC. Although the latency of VEP has been extensively studied, the results of these studies were contradictory across most VEP/VEFs observations [23]. The causes of the discrepancies were probably multifaceted, which may be largely accounted for by methodological differences (space frequency, reversal rate), patient’s state (time interval to nearest attack), and nosological diversity [36]. Since the prolongation of VEP/VEF latency was identifiable in other diseases, such as ophthalmopathy, stroke, Parkinson’s disease [37, 38], this phenomenon seemed not to be migraine-specific, and therefore may not be an ideal diagnostic tool for migraine. Notably, a diffidence of VIIs latency and spectrum power between the left and the right hemisphere was observed in migraine subjects when compared to HC. Early VEPs studies also reported the asymmetrical responses in migraine patients [23, 39]. It has been proposed that the susceptibility in different hemispheres of the migraine brain is heterogeneous. Unfortunately, the correlation between this phenomenon with the lateralization of pain was not analyzed in the current study because of the limited number of migraine subjects.

Our results from time-frequency analysis demonstrated that the activation pattern was similar between interictal migraine and HC in both low and high-frequency ranges. When compared to our previous studies using motor and auditory modality [29, 31], the VEFs showed a normal presence at low frequency but reduced presence at high-frequency range either on the sensor level or source level. It is known that high-frequency oscillations (HFOs) can be detected from event-evoked potentials using bandpass filters as evidenced by a considerable sum of electrophysiology studies [40–42]. In the present study, we used a wide frequency range because high-frequency oscillation has been proposed to be associated with abnormal thalamocortical activities [40, 41], which is a pivot mechanism in the inhibitory pathway in migraine. In concord with these results, the present study also showed a diminution of spectral power in the high-frequency range in migraineurs when compared to HC. As mentioned above, spectral power in this study involves the sum of all cortical activation power detected on the sensor level, thereby representing the entire cortical activation. Interestingly, the low source strength was low in the occipital lobe from the low-frequency range, which seems to be paradoxical with a significantly high RMS value in migraineurs. The contradiction may possibly be due to the difference between the two methodologies, that is, the spectral power may contain noise from other regions of the brain while the noise was filtered on the source level. In the high-frequency range, however, spectral power was consistent with source strength. This phenomenon supports the notion that low-frequency signals might be produced by a large brain area whereas high-frequency brain signals are more likely produced by specific regions of the brain, thus are highly localized and may provide precise spatial information regarding cortical dysfunction [33, 43].

Our data is in favor of an interictal hypo-activation condition of the visual cortex in interictal female migraineurs in both the low and high-frequency range. These findings are in line with many VEP/VEF studies, thereby indicating a low initial amplitude of VEP/VEF in interictal migraineurs [44, 45]. According to numerous electrophysiology studies, the excitability in the interictal migraine brain is better referred to as hyper-responsivity rather than hyperexcitability because the abnormality of the migraine brain involves primarily characteristics due to lack of habitation for a sensor stimulus [12, 36, 44, 46]. In this study, we alternatively focused on the spatial-frequency trait, and source strength of the visual-evolved neuromagnetic responses especially the major component (VIIs) instead of the habituation process. Our data on source strength showed that the initial activation of VEF was low compared to that of HC, which was in line with a low first VEP/VEF amplitude in other habitation studies. Low initial amplitude and a lack of habitation of event-related response (auditory, visual, somatosensory etc.) in migraineurs have been extensively reported in previous studies [13, 47, 48]. These reproducible abnormalities indicated a decreased pre-activation level of the cortical circle and increased neuronal hyperresponsivity in female migraineurs between attacks. High-frequency oscillations studies have shown that the hyperresponsivity and low pre-activation in interictal migraine are associated with a dysfunctional thalamocortical afferent pathway may potentially contributed by aminergic neurotransmitter disposition [38–40].

Regarding source location, the data shown there was not diffident from the odds of activation in other brain areas other than the primary visual cortex in both the low and high-frequency range between the two groups. Migraine may be associated with visual-related network dysfunction [49], therefore, our initial hypothesis of this dysfunction of visual cortex in the migraine brain will evoke more extensive visual to participant in the response of visual stimulus. However, taking together the low source power results observed in migraine subjects, the phenomenon could be partly understood. The relation between visual cortex and pain perception is intriguing although the visual cortex is conventionally considered to be outside of pain matrix. On the one hand, tonic pain can change the excitability of the visual cortex in HC [50]. In their study, healthy subjects showed normal VEP patterns during baseline and no-pain conditions, while during pain and after-effects of tonic pain condition they showed identical VEP patterns as interictal migraineurs (low initial VEP amplitude and lack of habituation). On the other hand, the visual cortex may exert influence on the perception of pain, a potential top-down inhibitory pathway from the visual areas to trigeminal-cervical nociceptors may participant in the modulation of pain [51]. Especially, intra-individual correlations between dysfunction of visual and pain perception regions were found in migraineurs but not in HC [52]. Given that migraine is associated with the abnormal condition of subcortical/intercortical inhibitory pathway [46, 53], the dual-directional regulation between visual cortex and pain will open a new window to the culprit of migraine.

In this study, a correlation between MEG results (latency, spectral power) and clinical parameters (i.e. intensity of headache, headache-history duration, frequency of headache attacks per month) was not observed, which is in line with data presented in many VEP/VEF studies [54]. Taken together, our finding supports the notion that subcortical/intercortical instead of cortical pathways were more likely to be the underlying mechanism for altered excitability in the visual cortex, which has been proposed by a large number of studies [1, 6]. In a recent study using high frequency domains, it was suggested that thalamocortical dysrhythmia was also attributed to functional changes in the migraine brain [41], however, the exact mechanism of subcortical dysfunction still needs to be determined.

## Limitation

Our study has some limitations. First, the sample size of HC is small, and patients with other subtypes of migraine (i.e. migraine with aura) were not enrolled in the study. Secondly, all participants are female, therefore the study needs to be extended to male migraineurs. Lastly, in this study, data from migraineurs during the ictal phase was not collected because participants may fail to cooperate during the scan with potential exacerbation by the visual stimulus. Therefore, a full picture of the periodic changes over the migraine cycle was not obtained in present study.

## Conclusions

Neurological responses following a visual stimulus were abnormal in female migraine patients between attacks when compared to HC. This difference was more significant in the high-frequency range.

## Abbreviations

cPVC: Contralateral primary visual cortex
RBcPVC: Regions beyond contralateral primary visual cortex
